# Order effects: a randomised study of three major cancer-specific quality of life instruments

**DOI:** 10.1186/1477-7525-3-37

**Published:** 2005-05-31

**Authors:** Yin-Bun Cheung, Celestine Lim, Cynthia Goh, Julian Thumboo, Joseph Wee

**Affiliations:** 1 MRC Tropical Epidemiology Group, London School of Hygiene and Tropical Medicine, London WC1E 7HT, UK; 2 Clinical Trials and Epidemiological Sciences, National Cancer Centre, 11 Hospital Drive, 169610, Singapore; 3 Department of Palliative Medicine, National Cancer Centre, 11 Hospital Drive, 169610, Singapore; 4 Department of Rheumatology and Immunology, Singapore General Hospital, Outram Road, 169608, Singapore

**Keywords:** health-related quality of life, cancer, order effect

## Abstract

**Background:**

In methodological studies and outcomes research, questionnaires often comprise several health-related quality of life (HRQoL) measures. Previous psychological studies have suggested that changing the sequential order of measurement scales within a questionnaire could alter the pattern of responses. Yet, information on the presence or absence of order effects on the assessment of HRQoL in cancer patients is limited.

**Methods:**

An incomplete block design was used in this study of 1277 cancer patients. Each patient filled out a questionnaire package that contained two of the three major cancer-specific HRQoL instruments, namely the Functional Assessment of Cancer Therapy – General, the European Organization for the Research and Treatment of Cancer Core Quality of Life Questionnaire and the Functional Living Index – Cancer. Within a questionnaire package the sequential order of the instruments contained were randomised. Measurement properties of the instruments, including the number of missing values, mean HRQoL scores, known-groups validity and internal consistency were compared between samples of different presentation orders.

**Results:**

No effect of presentation order on the four properties aforementioned was found.

**Conclusion:**

Presentation order is unlikely to alter the responses to these HRQoL instruments administered in cancer patients when any two of them are used together.

## Background

The order of questions in an interview may affect the responses to each question [1-3]. Conventional wisdom suggests that surveys should begin with simple, descriptive and non-sensitive questions [2,3]. The items used in composite measurement scales may also be subjected to a context effect [4]. Yet, there has been limited information in the area of quality of life research to confirm if the presentation ordering of composite measurement scales within a questionnaire would alter the results.

Jensen et al. [5] and Mook [6] discussed various reasons why order effects could appear. For instance, respondents may experience fatigue or lose concentration towards the end of a questionnaire and as a result, the probability of misinterpretation and omission of items may increase. According to this view, the strength of order effects is related to the length of the questionnaire. Moreover, respondents may produce different patterns of responses as the previous questionnaires desensitise or familiarize them with a topic.

The development of new health-related quality of life (HRQoL) instruments frequently employs multiple instruments in order to determine convergent and divergent validity. It is uncertain whether the validity or other measurement properties of an instrument could be affected by the presentation order. Furthermore, the possibility of an order effect points to the need for caution during the comparison of information across studies in which the HRQoL measurement scales are not presented in the same order, even if the questions are identical. It is therefore important to determine or prevent order effects in such situations.

Using randomised and counterbalanced designs, Jensen et al. [5] and Lucas [7] demonstrated that the presentation order of some psychological instruments had an impact on the scores. In a postal survey that included four health and HRQoL measurement scales, the researchers used two versions of a questionnaire [8]. One began with generic measures followed by specific measures; another presented the constituent groups of items in chronological order according to the time period the items referred to. They found that the questionnaires using chronological order were returned more promptly although the presentation order did not appear to affect the answers. One study investigated this issue in the assessment of the quality of life of cancer patients [9]. The participants self-administered a questionnaire in which the European Organisation for Research and Treatment of Cancer Core Quality of Life Questionnaire (EORTC QLQ-C30) preceded the Functional Assessment of Cancer Therapy – General (FACT-G). The two instruments contained similar questions on four aspects of HRQoL, namely, pain, nausea, meeting family needs, and general satisfaction. The investigators found that the four questions in the two instruments indicated similar level of HRQoL even though the patients had been exposed to the EORTC QLQ-C30 questions before they answered the similar FACT-G questions.

In a recent study, the FACT-G and Quick-FLIC (an abbreviated version of the Functional Living Index – Cancer, FLIC [10]) were used [11]. Alternating sequencing of these two HRQoL instruments were carried out to form two different questionnaire packages. The study showed that there was no major effect of presentation order on the mean scores, amount of missing values, and known-groups validity and internal consistency of the instruments. The inadequacies of the study were that it used a relatively short questionnaire as the mean time to complete was only 15.0 minutes; it involved only two HRQoL instruments; and the sample size was relatively small.

The present study aimed to verify the previous findings about the lack of order effects in the assessment of cancer patients' quality of life. It used a larger sample size and longer questionnaires that involved three major HRQoL instruments commonly used in oncology.

## Methods

### Design

This study used an incomplete block design [12], in which participants were randomised to receive one of the following six questionnaire packages (in this order of presentation): (1) EORTC QLQ-C30 and FACT-G, (2) FACT-G and EORTC QLQ-C30, (3) EORTC QLQ-C30 and FLIC, (4) FLIC and EORTC QLQ-C30, (5) FACT-G and FLIC, and (6) FLIC and FACT-G. We chose against using a complete block design of having each patient complete all three questionnaires because past experiences suggested that some patients might be unable or unwilling to spend so much time and concentration on it. In the current study, the mean time taken to complete the interview was 20.4 minutes, but the 90^th ^percentile was 39 minutes. Due to logistic considerations, the randomisation used days rather than individuals as units and assigned the six packages in blocks of six days [13]. In the examination of order effects on FACT-G, for instance, the FACT-G data from packages (2) and (5), where FACT-G was administered first, were compared against those from packages (1) and (6), where FACT-G was administered last. For brevity, we used the phrases order A and order B to mean an HRQoL instrument was administered first and last, respectively.

### Patient recruitment

Patients were recruited from the National Cancer Centre, Singapore, which serves about 70% of the cancer patients seen by the public sector of the country, from September 2003 to May 2004. The study was approved by the Ethics Committee of the Centre. Patients were approached while they were in the waiting areas of the specialist outpatient clinics, ambulatory treatment unit and the therapeutic radiology department of the Centre. The inclusion criteria were: literate in English or Chinese, aged 18 years or older, and agreeable to give written informed consent. The patients were heterogeneous in clinical profiles, such as having different types of tumour, and were fitting for the study of the three instruments that were designed for application to all cancer patients.

Singapore is a multi-ethnic society with the Chinese forming about 70% of the total population. The Chinese participants had the option of answering either an English or a Chinese questionnaire according to their lingual preference, whereas other participants answered an English questionnaire. Participants were requested to self-administer the questionnaire packages (where possible). Upon request by the patients, interviews would be administered by one of the two research coordinators of the project.

### Instruments

The FACT-G version 4 and EORTC QLQ-C30 version 3 were used. The FLIC had been modified in two aspects for use in Singapore [14,15]. Firstly, the word "cancer" was removed from the questions because some patients, particularly the older patients, might be unaware of their diagnosis and sometimes their families might not want them told the diagnosis. In this regard, it is of note that the FACT-G and EORTC QLQ-C30 do not mention the word cancer. Secondly, the visual analogue scale was difficult to some patients, especially the older and less educated. It was replaced by a seven-point Likert format scale. Similar modifications of the FLIC have also been reported in other countries [16,17].

The questionnaire packages each began with a page of demographic and health questions on information such as Eastern Cooperative Oncology Group (ECOG) performance status [18] and whether the patients were on chemotherapy and/or radiotherapy. Treatment status was classified as whether the patient was on chemotherapy and/or radiotherapy or not (yes or no).

### Statistical considerations

All HRQoL items were recoded such that a higher score reflects a better quality of life. Missing values in the FACT-G, FLIC and EORTC QLQ-C30 were imputed by the half-rule [19]. ANOVA and Chi-square tests were used to compare continuous and categorical variables, respectively, between patients who answered the six questionnaire packages. Fisher's exact test was used to compare the number of missing values in each instrument between orders of presentation. Negative binomial regression was used to estimate the difference in mean number of missing values between presentation orders and the confidence interval (CI) [20]; linear regression was used for HRQoL scores. Cronbach's alpha was calculated for each HRQoL instrument in each order of presentation. There is no established analytic procedure for the estimation of CI for the difference in Cronbach's alpha. We employed the bootstrapping method, with 1000 replications [21].

In line with commonly accepted practice for equivalence studies, 90% confidence intervals (CI) were estimated [22,23]. Equivalence was declared if the 90% CI fell totally within an equivalence zone. For the comparison of mean number of missing values, the equivalence zone was pre-defined as ± 1 item. For the comparison of Cronbach's alpha, the zone was ± 0.1.

There is no consensus to the definition of equivalent HRQoL scores. Using various clinical criteria, Cella et al. [24] suggested that the minimal clinically significant difference on the FACT-G scale is 4 points. Based on the assessment of subjective significance, Osoba et al. [25] suggested that "a little" change on the EORTC QLQ-C30 global quality of life scales was approximately 5 to 10 points, on a scale of 0 to 100. Interestingly, both studies approximately agreed with Cohen's [26] suggestion that an effect size between 0.2 to less than 0.5 standard deviation (SD) is small. In the present data set, 4 points of the FACT-G score and 5 points of the EORTC global functioning score are equivalent to 0.25 and 0.23 of their SD's. Therefore we defined an equivalence margin as ± 0.25 SD, rounded to the nearest integer. It corresponded to 4, 6 and 5 points for the FACT-G, FLIC and EORTC QLQ-C30, respectively. Furthermore, we defined a "small difference" margin as ± 0.5 SD. This took into account Osoba et al. [25] about a little change (10 points). The small difference margins for the FACT-G, FLIC and EORTC QLQ-C30 were 8, 12 and 10 points, respectively.

The main analyses did not adjust for covariates. Supplementary analyses adjusted for covariates shown in table 1 using the multiple regression analysis approach.

**Table 1:** Respondent characteristics by questionnaire package (N = 1277)

**Variable**		**Questionnaire Package**						
			
		EORTC QLQ-C30 + FACT-G (N = 200)	FACT-G + EORTC QLQ-C30(N = 240)	EORTC QLQ-C30 + FLIC (N = 233)	FLIC + EORTC QLQ-C30 (N = 188)	FACT-G + FLIC (N = 215)	FLIC + FACT-G (N = 201)	*p*-value^(a)^
Age	Mean (SD)	51.0 (12.0)	51.6 (12.5)	50.6 (9.9)	51.0 (10.3)	51.4 (10.5)	51.4 (11.6)	0.933
								
Gender	Male	43.5%	40.8%	36.1%	41.0%	43.7%	38.3%	0.547
	Female	56.5%	59.2%	64.0%	59.0%	56.3%	61.7%	
								
Race	Chinese	88.0%	91.3%	90.6%	91.0%	90.7%	89.1%	0.446
	Malay	6.5%	4.6%	3.4%	2.7%	3.7%	4.5%	
	Indian	4.0%	3.8%	3.0%	4.3%	2.3%	5.5%	
	Others	1.5%	0.4%	3.0%	2.1%	3.3%	1.0%	
								
Education	Primary or below	22.1%	20.4%	21.0%	19.2%	23.8%	21.9%	0.212
	Secondary	44.7%	43.8%	49.4%	57.9%	45.3%	47.8%	
	Post-secondary	33.2%	35.8%	29.6%	22.9%	30.8%	30.4%	
								
ECOG	0–1	75.5%	72.5%	75.1%	71.8%	69.8%	72.0%	0.772
	2–4	24.5%	27.5%	24.9%	28.2%	30.2%	28.0%	
								
Treatment	Inactive	59.0%	69.6%	59.2%	61.7%	60.9%	62.7%	0.186
	Active	41.0%	30.4%	40.8%	38.3%	39.1%	37.3%	
								
Tumor	Breast	30.5%	33.8%	40.8%	33.0%	33.0%	34.8%	0.719
	Lung	7.5%	8.3%	5.6%	6.4%	9.8%	10.0%	
	Colo-rectal	14.5%	10.8%	12.0%	13.8%	13.5%	11.9%	
	Gynaecological	5.0%	5.8%	3.4%	9.0%	4.7%	6.5%	
	Nasopharyngeal	13.5%	13.3%	12.5%	13.3%	14.4%	16.4%	
	Head & Neck	8.0%	5.8%	5.2%	5.9%	5.1%	7.00%	
	Others	21.0%	22.1%	20.6%	18.6%	19.5%	13.4%	
								
Self-administered	Yes	76.5%	79.6%	76.8%	75.5%	75.4%	73.1%	0.738
	No	23.5%	20.4%	23.2%	24.5%	24.7%	26.9%	
								
Language	English	57.5%	56.7%	49.8%	51.1%	60.0%	57.2%	0.219
	Chinese	42.5%	43.3%	50.2%	48.9%	40.0%	42.8%	

(a) Difference between six questionnaire packages tested by ANOVA for age and Chi-square for categorical variables.

A sample size of 270 per instrument per order of presentation would give a power of 80% and a 5% probability of the type I error for confirming equivalence (± 0.25 SD) between different orders of presentation [22]. The sample size here was about 50% larger because the primary purpose of the study (to compare the variability of the different HRQoL instruments [27]) required it.

### Results

A total of 1317 patients consented to participate. Some patients' family members insisted on completing the questionnaire on their behalf. These proxy interviews were excluded. After this exclusion the number of subjects was 1277.

Table 1 provides a descriptive summary of the background characteristics of the patients by questionnaire package. The six groups of patients were similar in clinical and demographic characteristics (each p > 0.10). They were also similar in terms of mode of administration of the questionnaires and the language used (each p > 0.10).

Table 2 shows the number of missing values in the FACT-G, FLIC and EORTC QLQ-C30 by presentation order. The Fisher's exact test showed no significant differences in the distribution of the number of missing values between the two presentation orders A and B for the three instruments (each p > 0.10). The mean number of missing FACT-G item values was 0.03 higher (90% CI = -0.11 to 0.18) among patients who answered the FACT-G first than those who answered the FACT-G second. The corresponding figures for the FLIC and EORTC QLQ-C30 were 0.01 (-0.11 to 0.13) and 0.11 (0.04 to 0.18), respectively. All three confidence intervals totally fell within the pre-defined equivalence zone of ± 1 item. Further analysis using multiple regression analysis to adjust for the covariates shown in table 1 gave similar results. The mean difference (90% CI) between presentation orders in FACT-G, FLIC and EORTC QLQ-C30 missing items were, respectively, 0.04 (-0.10 to 0.18), -0.09 (-0.27 to 0.09) and 0.16 (0.04 to 0.28).

**Table 2:** Number of missing values in FACT-G, FLIC and EORTC QLQ-C30 by presentation order^(a)(b)^

	**FACT-G**		**FLIC**		**EORTC QLQ-C30**	
**Number of missing values**	Order A (N = 455)	Order B (N = 401)	Order A (N = 389)	Order B (N = 448)	Order A (N = 433)	Order B (N = 428)
0	207	195	303	345	368	380
1	157	139	47	52	46	40
2	48	36	16	27	12	6
3	20	9	15	16	2	0
4	9	8	4	3	3	2
5	5	4	0	4	1	0
6	4	3	1	1	0	0
7	2	2	1	0	0	0
8	3	2	1	0	0	0
≥9	0	3	1	0	1	0
Mean	0.96	0.93	0.44	0.43	0.25	0.14
p-value (Fisher's exact test)	0.825		0.457		0.308	

(a) Order A and order B mean, respectively, the HRQoL instrument was placed first and second in the questionnaire.

(b) The FACT-G comprises 27 items, the FLIC comprises 22, and the EORTC QLQ-C30 comprises 30 items. Therefore the results are not comparable across questionnaires.

Table 3 shows the means and standard deviations of the FACT-G, FLIC and EORTC QLQ-C30 total / global functioning scores according to order of presentation. The means and standard deviations were similar between the two orders across all three instruments. The mean FACT-G score was 2.44 points higher in the interviews where FACT-G was administered first. The 90% CI was 0.66 to 4.24, slightly exceeding the pre-defined equivalence zone of ± 4 points but not exceeding the "small difference" zone. The means of FLIC scores were almost identical in the two presentation orders and the confidence interval totally fell within the pre-defined equivalence zone of ± 6 points (difference = -0.62; 90% CI = -3.20 to 1.97). The mean EORTC QLQ-C30 score was 3.43 points lower in interviews where the EORTC QLQ-C30 was administered first; the confidence interval (-5.87 to -0.99) slightly exceeded the pre-defined equivalence zone of 5 points but not the "small difference" zone. The results after adjustment for the covariates in table 1 were similar. The mean difference (90% CI) between presentation orders in FACT-G, FLIC and EORTC QLQ-C30 scores were, respectively, 2.78 (1.28 to 4.28), 0.28 (-1.77 to 2.33) and -4.16 (-6.27 to -2.05).

**Table 3:** Comparison of FACT-G, FLIC and EORTC QLQ-C30 total/global functioning scores by presentation order^(a)^

	**FACT-G**		**FLIC**		**EORTC QLQ-C30**	
	Order A (N = 445)	Order B (N = 390)	Order A (N = 378)	Order B (N = 436)	Order A (N = 433)	Order B (N = 428)
Mean	85.92	83.48	123.56	124.18	63.68	67.11
Difference in means (95% CI)	2.44 (0.66 to 4.24)		-0.62 (-3.20 to 1.97)		-3.43 (-5.87 to -0.99)	
SD	15.44	15.94	21.84	22.79	21.69	21.83

^(a)^Order A and order B mean, respectively, the HRQoL instrument was placed first and second in the questionnaire.

Table 4 presents the mean values of the FACT-G, FLIC and EORTC QLQ-C30 total / global functioning scores by performance status and presentation order. All three instruments indicated a statistically significantly poorer quality of life in patients who had a poorer performance status (ECOG score 2 to 4) regardless of presentation order (each p < 0.05). In the case where FACT-G was administered first, the FACT-G score was 9.39 points higher in patients with better performance status. In the case where it was administered last, the FACT-G score was 6.68 points higher in such patients. The difference between the two estimates of between-group difference was 9.39 – 6.68 = 2.71 (90% CI = -1.83 to 7.24). Similarly, the differences (90% CI's) in between-group difference for FLIC and EORTC QLQ-C30 were 1.00 (-5.88 to 7.88) and -3.60 (-9.70 to 2.50), respectively. All three estimates of difference in between-group difference were within the equivalence zone of ± 0.25 SD. Although the three confidence intervals slightly exceeded the equivalence zone of ± 0.25 SD, they fell within the "small difference" zone of ± 0.5 SD. Again, adjustment for covariates did not make any practical difference. The difference between the two estimates of between-group difference for FACT-G, FLIC and EORTC were, respectively, 3.26 (-1.18 to 7.71), -0.79 (-7.14 to 5.56) and -3.58 (-9.47 to 2.32).

**Table 4:** Comparison of the mean FACT-G, FLIC and EORTC QLQ-C30 total/global functioning scores by ECOG score and presentation order^(a)^

**Order**	**ECOG score**	**FACT-G**	**FLIC**	**EORTC QLQ-C30**
**A**	0–1	83.93	119.37	58.73
	2–4	74.54	110.11	52.18
Difference in means		9.39 (p < 0.001)	9.26 (p = 0.002)	6.55 (p = 0.014)

**B**	0–1	80.64	118.39	64.21
	2–4	73.96	110.13	54.06
Difference in means		6.69 (p = 0.001)	8.26 (p = 0.004)	10.15 (p < 0.001)

Difference in A – difference in B		2.70	1.00	-3.60
90% CI		(-1.83 to 7.24)	(-5.88 to 7.88)	(-9.70 to 2.50)

(a) Order A and order B mean, respectively, the HRQoL instrument was placed first and second in the questionnaire.

Table 5 shows the Cronbach's alpha values of the three instruments by presentation order. The values were very similar across presentation orders and all three confidence intervals totally fell within the pre-defined equivalence zone of ± 0.1.

**Table 5:** Internal inconsistency of Fact-G, FLIC and EORTC QLQ-C30 by presentation order^(a)^

**Order**	**FACT-G**	**FLIC**	**EORTC QLQ-C30**
A	0.909	0.925	0.896
B	0.911	0.934	0.866
Difference in alpha	-0.002	-0.009	0.030
(90% CI)	(-0.022 to 0.099)	(-0.024 to 0.006)	(-0.024 to 0.082)

(a) Order A and order B mean, respectively, the HRQoL instrument was placed first and second in the questionnaire

## Discussion

Experimental evidence on the issue of order effects in the assessment of cancer patients' quality of life is scarce. There is substantial evidence that the measurement of psychological health and psychiatric morbidity are affected by the order of presentation of instruments [5-7]. However, a recent experimental study of 190 cancer patients suggested that the FACT-G and Quick-FLIC were free from such effects [11]. The researchers suggested that questions on HRQoL are less stigmatising and less threatening than questions about psychological problems. They also suggested that the more complicated skip patterns of some psychological / psychiatric measures may give rise to order effects related to the "punishment hypothesis" and "learning hypothesis" and that such patterns are rarely seen in cancer quality of life questionnaires [5,11]. In the present study of a substantially larger sample, we examined three HRQoL instruments commonly used in cancer research. Due to logistic considerations, we chose to use days rather than patients as the units of randomisation. We can think of no reason why bias should arise from this allocation scheme. Comparison of various background characteristics attested to the comparability of the patients randomised to different questionnaire packages. Secondary analyses adjusted for covariates gave similar results. The strength of order effects (if any) may depend on the length of questionnaire. The mean time to completion of the questionnaire packages was 20.4 minutes in the present study, about 5 minutes longer than that of the previous study, and the 90^th ^percentile was 39.0 minutes.

Our findings lend additional support to the previous finding that the order of presentation has little influence over the assessment of quality of life in cancer patients, evidenced by the following results. First, equivalence in the number of missing values and internal consistency of all three instruments across presentation orders was confirmed. Second, the mean values of the FLIC administered in different orders were also equivalent. The FACT-G and EORTC QLQ-C30 administered in different orders also showed similar mean values, although the confidence intervals of the difference slightly excluded the equivalence margin. Since the confidence intervals totally fell within the "small difference" zone of ± 0.5 SD, it can be concluded that at most the order of presentation has a small effect on mean FACT-G and EORTC QLQ-C30 global scores. Third, regardless of presentation order, all three instruments revealed a statistically significant difference in quality of life between patients with better versus poorer performance status. Again, the confidence intervals totally fell within the pre-defined "small difference" zones although not the equivalence zones. Known-groups validity did not seem to be affected. The use of multiple instruments in an interview is a common practice. The findings here should be good news for quality of life researchers as they suggest that previous studies were probably not unduly influenced by different ordering of instruments and that more complicated designs to prevent an order effect are not necessary.

The study of equivalence is often controversial. There is no clear-cut ground for the definition of equivalence. Still, studies reviewed above seem to converge to the conclusion that a difference smaller than 0.25 SD is irrelevant and 0.5 SD is small. Hence the ways we defined the equivalence and small difference zones. Secondly, the use of 90% CI is mainly a matter of common practice in equivalence studies rather than a matter of theoretical justification. In a discussion about the use of confidence intervals in equivalence trials, Senn [23] maintained that "all standards of significance and confidence are in any case arbitrary... little can be done to remove the arbitrary element". Since both 90% and 95% are arbitrary, it is our preference to adopt the common practice of using 90% CI. The response rate to this study was about 60%. Though this is not a high response rate, the findings are relevant because patients who refused to participate in the assessment of quality of life were not the concern of the present study. Whether there is an order effect in questionnaire presentation does not have any relevance to patients who do not participate, and vice versa. One limitation of the present study was that, although the point estimates seemed to suggest the lack of an order effect in mean scores and know-groups validity, some of the confidence intervals concerned slightly stretched across the equivalence margins. As such, a more definite conclusion awaits further studies. Moreover, the issue should be assessed again if the interviews concerned are considerably lengthier than the present one.

## Conclusion

There is no evidence of any major impact of the order of presentation on the assessment of cancer patients' quality of life when two of the three questionnaires – FLIC, FACT-G and EORTC QLQ-C30 – are used together.

## Authors' contributions

YBC conceived of the study, participated in the experimental design, developed the statistical framework, carried out part of the statistical analysis, and drafted part of the manuscript. CL conducted a large part of the statistical analysis and drafted part of the manuscript. CG participated in the research design, and the interpretation and discussion of findings. JT participated in the research design, the development of the statistical framework for the equivalence analysis, the interpretation of findings, and writing of the manuscript. JW participated in the research design, and the interpretation and discussion of findings. All authors read and approved the final manuscript.
